# Exploring the Role of the Advanced Nurse Practitioner (ANP) Older Persons From the Perspective of the Interdisciplinary Team

**DOI:** 10.1155/jonm/5522550

**Published:** 2026-03-25

**Authors:** A. Mullally, M. Doolan, C. Brennan, L. Burke, A. Farrelly, G. Flynn, K. Heduvan, G. Keena, O. Lynch, J. Mannion, F. Moore, M. Nolan, M. McDonnell-Naughton

**Affiliations:** ^1^ Department of Nursing and Healthcare, Technological University of the Shannon Midlands Midwest, Athlone, Ireland; ^2^ Department of Nursing, Health Services Executive Integrated Health Area, Midlands, Ireland

## Abstract

**Background:**

Advanced Nursing Practice is well established in Irish healthcare; these nurses are educated to practice at higher levels of competency as Advanced Nurse Practitioners (ANPs). Nurses working as ANP Older Persons are relatively new and offer a unique skillset that facilitates the delivery of holistic person‐centred care. To date, this represents novel research, as the ANP role in Older Persons’ care has not previously been explored from the perspective of the interdisciplinary team.

**Aim:**

The aim of this research was exploring the role of the ANP Older Persons from the perspective of the interdisciplinary team. The objective of this study was to gain a greater insight of the IDT’s perception of the ANP Older Persons’ role.

**Methods:**

A total of 40 participants were interviewed in 11 focus groups using semistructured interviews. Participants included representation from health and social care professionals, nurses and medical staff.

**Findings:**

Data were collected and analysed using Braun and Clarke (2021) theoretical framework, and results are presented in three themes: *Contribution of the ANP Older Persons’ Role to Clinical Practice and Patient Care*, *Contribution of the ANP Older Persons’ Role to the Interdisciplinary Team* and *Barriers to the Development of the ANP Older Persons Role* are the three emerging themes.

**Conclusion:**

The findings indicate that the ANP Older Persons is a valuable resource in the IDT providing education, leadership, clinical expertise and enhancing patient care. Ambiguity exists around defining advanced practice nursing in older person’s services, and there are barriers to the development of the ANP Older Persons’ role.

## 1. Background

The International Council of Nurses​ [1] defines an Advanced Nurse Practitioner (ANP) as “a generalist or specialised nurse who has acquired, through additional graduate education (minimum of a master’s degree), the expert knowledge base, complex decision‐making skills and clinical competencies for Advanced Nursing Practice, the characteristics of which are shaped by the context in which they are credentialed to practice.” Carney et al. [2] describes Advanced Nurse Practice as a nursing role that exceeds all previous boundaries grounded in the nursing field, unique in comparison to all other branches within nursing.

In Ireland, however, the concept of Advanced Nurse Practice originated as a career progression pathway following recommendations from the Commission on Nursing [3], rather than as a theoretical evolution of nursing practice [4]. This was further advanced through national policy, most notably the Department of Health [5] policy on the development of ANP posts, which aimed to enhance service delivery and reduce waiting times in priority areas including older person services.

The Nursing and Midwifery Board of Ireland [6] defines the ANP role as practicing at an advanced level, providing full, holistic, episodic care within their clinical speciality. ANPs demonstrate critical clinical decision‐making skills, with multiple competencies that enable them to work as independent practitioners. However, the ANP role continues to vary in terms of scope of practice, autonomy and regulation internationally [2, 6, 7].

To qualify, registered nurses in Ireland must complete a Master of Science (MSc) degree and demonstrate practice at a higher level of capability, autonomy and clinical expertise [6]. Their practice is governed by The Advanced Practice (Nursing) Standards and Requirements, which outline six domains of practice: professional values and conduct, clinical decision‐making, cognitive and knowledge competencies, communication, team management and leadership and professional scholarship (Appendix I).

The ANP in Older Persons is a relatively new development within advanced nursing practice in Ireland. This speciality has emerged in response to demographic challenges, particularly increasing life expectancy, multimorbidity and the growing complexity of care needs among older adults. While the ANP’s role is well‐established in areas such as emergency care and chronic disease, it is less developed in older person services. The Department of Health’s policy pathway to ANP [5] prioritised expansion in this area to improve quality and access to care, while promoting interdisciplinary integration and clinical leadership.

Despite the proliferation of ANP roles, particularly in older person services, there remains limited published evaluation of their integration into existing health systems or their alignment with the broader health policy agenda (see [8]). Leadership, integration and governance are core domains of the ANP role. However, these elements have been insufficiently explored in national research, particularly regarding interprofessional perceptions. The international literature also highlights the need for structured evaluation of role implementation and system‐level impact.

A review of the literature between 2009 and 2023 illustrates a significant dearth of research on the ANP Older Persons’ role, particularly from the perspective of interdisciplinary colleagues. Studies by Thompson and McNamara [9] and Carney et al. [2] underscore a persistent lack of awareness about ANP role scope, governance structures and leadership responsibilities, which may lead to underutilisation and reduced effectiveness in practice. Furthermore, the lack of defined role expectations across settings can hinder both integration and evaluation efforts.

This study was informed by a constructivist paradigm and underpinned by Braun and Clarke’s [10] reflexive thematic analysis, which acknowledges researcher subjectivity and coconstruction of meaning within the data. This approach allowed exploration of interdisciplinary perspectives through a lens of professional identity and collaboration, offering insight into how advanced practice roles are interpreted and negotiated within complex healthcare systems.

This research aimed to address these knowledge gaps by exploring the ANP Older Persons’ role through the lens of the interdisciplinary team (IDT). Gaining deeper insight into how IDT members perceive and understand the ANP Older Persons’ role can help identify both facilitators and barriers to successful integration, collaboration and impact on Older Person’s care.

## 2. Methodology

### 2.1. Design and Setting

The purpose of this qualitative study is to explore the ANP Older Persons’ role from the perspectives of the IDT, specifically within the Midlands area in Ireland. This research study was conducted over a period of months (December 2023–July 2024) and employed a qualitative descriptive design as first employed by Sandelowski [11], with data analysed using Braun and Clarke’s [12] reflexive thematic analysis. While the qualitative approach is known as the least theoretical within qualitative research designs, it allowed for the full exploration of the participants’ opinions and explanations of the role, capturing similarities and differences within the data [13]. This design was appropriate for capturing interdisciplinary perceptions, which are often shaped by expectations, observed outcomes and clarity of role boundaries.

### 2.2. Sampling and Recruitment

Purposeful sampling facilitated the recruiting of participants from the IDT across four chosen sites within the Irish Midlands area. All participants were required to have experience working with an ANP Older Person to be eligible for inclusion. The study was advertised in all clinical sites. Advertisement posters contained information about the study and how to participate. Posters included QR codes for staff to register and attend the focus groups. Walk‐in participants were also permitted. Information regarding the study was circulated via work email and verbally within the workplace.

Participants were required to meet two inclusion criteria: working as part of the IDT in the chosen clinical sites and working alongside an ANP Older Person. An attempt was made to ensure a mix of professional backgrounds in each focus group; however, due to voluntary participation and scheduling constraints, some variation in group composition occurred. The sample may overrepresent individuals with more established or positive working relationship with the ANP Older Persons. All participants were provided with detailed information about the study and gave verbal and written consent prior to participation. They were informed that their identity would remain anonymous and that they could withdraw from the study at any stage, up to 2 weeks poststudy.

### 2.3. Data Collection

The researcher facilitated all focus groups, with a moderator present. Focus groups were chosen for this study as they facilitate discussion, idea sharing and debate within a group. The broad range of qualitative interviews facilitates the exploration of the participants on an emotional and honest, in‐depth level [14]. In addition, focus groups gather valuable insights from a specific group of people and allow the researcher to gain a deeper understanding of participants’ thoughts and beliefs [15]. This form of interview can be especially beneficial for the researcher when there is a lack of information on the topic being studied [16, 17].

Creating an appropriate environment to facilitate focus groups was important for the study as it allowed for participants to voice their opinions from the perspective of the IDT regarding the role of the ANP. A semistructured topic guide was used to ensure consistency across focus groups (Appendix III). A topic guide was created by the researcher to provide structure to the focus groups and provide purposeful questioning to the participants prior to the commencement of the study focus groups.

Although participants worked with ANPs Older Persons, it should be noted that ANPs may have had slightly different job descriptions or governance structures depending on the site. However, all were required to meet the core criteria outlined in the NMBI [6] Advanced Practice Standards and Requirements.

### 2.4. Ethical Considerations

Ethical approval was obtained from the Research Ethics Committee at Technological University of the Shannon Midlands/Midwest (TUS) and from the HSE Research Ethics Committee (RREC) for the Midlands Area and Corporate Division (HSE Dublin and Midlands). This study was conducted in full compliance with their regulations and terms of agreement. Informed consent was obtained from all participants, and anonymity was maintained using codes and pseudonyms. Consent was an ongoing process throughout the study. No incentives were offered for participation.

### 2.5. Data Analysis

Data collected from the focus groups were transcribed and analysed using Braun and Clarke’s [12] framework. Reflexive thematic analysis was used as an analytic method within a qualitative descriptive design, allowing rich description while acknowledging researcher reflexivity. NVivo [18] qualitative data analysis software was utilised to assist the researcher in recording data, grouping codes and mapping the categorisation of themes formed. Content highlighted as important or relative to the researcher was initially grouped broadly. This condensed the data into units of common content. Inductive coding commenced after units of data was created. This process categorised data at a descriptive level and filtered out irrelevant content. Codes were then developed and compared against each other, identifying similar and contrasting features. The researcher categorised this process through creating initial codes, subcodes and final themes. Data collection continued until thematic sufficiency was achieved, with no new themes emerging in later focus groups. The sample size was considered adequate to capture interdisciplinary perspectives across sites. Appendix II presents the table of the coding framework used in the analysis.

### 2.6. Reporting Standards

This qualitative study is reported in accordance with the Consolidated Criteria for Reporting Qualitative Research (COREQ). Reporting decisions were guided by best practice standards for focus group research.

## 3. Results

Participants (*n* = 40) took part in 11 separate focus groups. Each focus group was composed of a professional mix of IDT members, with an aim to include a diverse representation across all groups. The professional composition of groups varied. All groups included at least three different disciplines to ensure multiperspective discussions. Table 1 shows the participant numbers by discipline.

**TABLE 1:** Participants by discipline.

Occupation	Number of participants
Physiotherapist	7
Dietician	3
Occupational therapists	5
Nurse managers	3
Nurse specialists	8
Candidate ANP	1
Staff nurses	2
Consultants	4
Directors of nursing	2
Speech and language therapists	3
Cardiac physiologist	1
Healthcare assistant	1

Following data analysis under the structure of thematic analysis [12], three themes emerged, (i) Contribution of the ANP Older Persons’ role to clinical practice and patient care, (ii) Contribution of the ANP Older Persons’ role to the IDT and (iii) Barriers to the development of the ANP Older Persons’ role. Themes did not significantly differ across groups, though some nuances emerged based on specific professional roles, particularly among nursing staff versus allied health professionals.

### 3.1. Theme 1: Contribution of the ANP Older Persons’ Role to Clinical Practice and Patient Care

This theme focused on the contribution of the ANP to clinical practice and patient care and included three sub themes: prescribing; education, knowledge, skillset; and improved patient care. Participants shared numerous examples of how the ANP contributed to clinical practice in the above areas.

#### 3.1.1. Prescribing Role

Participants shared many exemplars of the ANP Older Persons’ ability to prescribe and deprescribe medicinal products, affirming that the ANP Older Persons are highly skilled and knowledgeable of patients’ medication. Participants highlighted the benefits of having an ANP Older Persons working alongside them when it comes to medication

What′s really useful when our ANP is there with us on the ground in ED is definitely around medications… (…) with deprescribing, they have been very helpful. (P2)

Without the ANP, you don’t have that medication management piece. (P4)

Her knowledge on, say, polypharmacy or dementia or delirium is huge. (P7)

#### 3.1.2. Education, Knowledge and Skillset Role

Participants described the ANP Older Persons’ willingness to share knowledge, and they provided examples where the ANP delivered education to the IDT. The ANP was described as a *“fountain of knowledge”* (P3) and is someone from whom the IDT *“learned a lot”* (P38).

Participants articulated the ANP Older Person’s capacity to implement *“recent evidence”* (P24) and best clinical practice into daily work. ANPs provide a wealth of knowledge and education for the IDT;

They’d keep me up to date and then they would do education and training as well. (P5)

Participants spoke about the skillset and overall older person knowledge and expertise that the ANP Older Persons brings, making them an asset to the IDT:

that piece of the puzzle that’s often missing… They always share knowledge and share skills. (P4)

It’s an aging population, so their skillset and knowledge is going to be more and more demand for better care. (P29)

The ANP Older Persons were described as *“someone who could identify the whole picture”* (P40) in examples such as *“handling family meetings, complex discharge and advanced care planning”* (P12). Participants in this study confirm that the ANP role is holistic and person‐centred:

They (ANP) can piece it all together, the whole framework and the nursing background, and then holistically, they see the patient. (P12)

One participant perceived the ANP Older Persons role as someone who *“does it all”* (P40)

Participants felt that given the ageing population, the demand for specialised care and scarcity of doctors, the ANP Older Persons would have the knowledge and expertise to tackle many of the ageing population’s needs;

Just overall older person knowledge and the experience that she has is amazing… she’ll always bring a lot to the discussion. (P2)

#### 3.1.3. Improved Patient Care

Participants clearly articulate the positive impact that ANPs have on patient care and perceive that ANPs (Older Persons) advocate for patients while considering the patient’s bio–psych–social needs:

Older people, they’re not often sick, like quite often, they just have one small episode and then they can go back to being as normal as they were before. But like if you could tap into ANPs for it to be that link, it would be perfect. (P34)

I think holistically, she’ll just look at the whole patient and put the patient first. (P12)

I think it’s a better quality of care for the patient at the end of it. (P4)

Examples where the ANP Older Persons reduced waiting time in ED and enabled the patient to be seen quickly and appropriately were cited by participants as improving patient care;

The ANP role is just amazing when you can get in and out of A&E in an hour and a half. It’s heaven, so it is. (P11)

P35 explains how an ANP Older Person will become a vital member of the IDT and is a necessity for the Older People’s care:

We have an ageing population. So really, especially in dementia, you know, the next 30 years it will be triple. So, if you look at resources that we have, you know, doctors are getting very limited. So, an ANP would be a good level of expertise that we would be able to tackle a lot of the issues for the ageing patients. (P35)

### 3.2. Theme 2. Contribution of the ANP Older Persons’ Role to the IDT

This theme highlights how the ANP Older Persons has contributed greatly to the IDT, according to the participants. The three subthemes discussed were Collaboration between the ANP Older Persons and IDT, Communication with the IDT and Leadership.

#### 3.2.1. Collaboration Between the ANP Older Persons and IDT

The workplace relationship became a central thread that fed into all aspects of the ANP Older Persons’ role and the IDT’s perception of this. Participants expressed strong, positive emotions about their relationship with the ANP Older Persons. Participants expressed the ANP’s ability to *“link between the medical and the nursing team”* (P7) due to the close relationship. The participants highly respect the ANP role and rely on them, saying that the ANP is the *“first person to call”* (P11).

She’s always available, very‐very approachable. (P12)

We have a good relationship and we’d be able to pick up the phone and say, look what do you think here? (P4)

The participants expressed having a confident understanding of the role due to a well‐established work relationship in the IDT. The ANP’s ability to maintain a healthy work relationship with colleagues reduced the likelihood of confusion surrounding the ANP Older Persons’ role:

You establish this working relationship with her because she’s there regularly. People know exactly what she’s doing. (P35)

The IDT felt that they could easily access the ANP Older Persons, when necessary, through a well‐established referral system setup within some of the sites investigated:

They have, like, their inclusion criteria and stuff that we can look up on their referral forms and stuff, so it’s very easy to, if we wanted to refer to them. (P25)

If you wanted to, I suppose, bounce something off someone, you could tend to go to her (ANP) first before you even go to a near a geriatrician because she’s as good if not better. (P34)

I suppose, you know, things that I might not have thought of or kind of new initiatives or just new things, you know, because that’s the ANP’s role. (P5)

The participants spoke about their reliance on the ANP Older Persons due to their knowledge and open demeanour. The IDT stated on many occasions how the ANP’s ability to make connections with others may be the reason behind the success of their role so far.

If you have an idea of where you want to go with a plan, she can maybe reassure that and back that up and kind of you know, bring it to the next level with what she thinks we need to do to get there. (P3)

If I have a concern about something, my ANP can either escalate it and act as a catalyst and support me in that or can also do the opposite and can help extinguish it. (P16)

I never doubt her competency or incompetency. (P12)

They would have definitely done some education with me on their role and how we can collaborate with the patient’s best interest and to see the overlap among the disciplines. (P16)

#### 3.2.2. Communication With the IDT

The ANP Older Persons are described as a skilful and effective communicator with the IDT expressing their appreciation for the regular check‐ins and the ANP’s ability to understand all professions at their level. Participants described the ANP as a *“link person”* and someone who is their *“first port of call”* (P2).

The communication and the relationship that everybody has with her like she’s just very open and welcoming. (P37)

We’re in touch nearly every day now at his stage. (P11)

A lot of messages go back and forth during the day in relation to the admissions and discharges. (P15)

The ANP was a really good support to me and she kind of guided me in how to set up my service and things like having inclusion and exclusion criteria and things like that. (P32)

In this study, participants displayed an understanding of how vast of a workload an ANP Older Person may have and their capabilities due to effective communication with the IDT. The ANP Older Person can link in with any IDT member and through effective communication and can assist other professions:

I was very impressed with her report, which saved me a lot of time… I would say I think it would be a bonus and a benefit to have the ANP. (P36)

It’s just their clinical knowledge really, their expertise, their communication and it’s a lot easier to access than a consultant or doctor. (P35)

#### 3.2.3. Leadership and Advancements in Nursing

Participants who were nurses at different levels expressed a significant increase in job satisfaction and enthusiasm within the nursing field. Participant P11 states that they are *“excited about nursing again because of what I have learned from the ANP”.* Nurses described the ANP as an influential clinical leader who fosters innovation, mentors junior staff and facilitates service improvements. Participants expressed that the presence of ANPs in clinical environments has advanced nursing leadership, strengthened interdisciplinary trust and encouraged professional growth. Other healthcare workers have observed these changes too:

My impression is that in the hierarchy of things, your consultants and doctors are almost relieved to have that ANP role there. (P17)

They’re one of my primary referrers and they’re just a great person to collaborate and liase with. (P16)

The team collegiality was very evident. The multifaceted aspect of the ANP Older Persons’ role is highly valued by the participants:

We all work really well together. (P4)

From my point of view, we are very interlinked and overlapping as well, so we work quite closely. (PT6)

She’s very good for linking up with therapists as well. (PT4)

Advancements within the nursing field such as the role of the ANP Older Persons has changed how nursing is perceived by the IDT. Participants outside the nursing field, such as P36, P1 and P17, have highlighted the benefits of accelerating nursing and breaking down professional boundaries. Participants suggested that the ANP provides expert knowledge and is able to lead clinical care through education and support for colleagues:

“She is very much a leader” (P34)

“She helped set up a pre‐admission document using her paperwork, my paperwork and the nursing care plan and we’re constantly tweaking it”. (P11)

“Having her there has extended the network. I am now comfortable picking up the phone and ringing one of the geriatricians about one of our patients, which is something I never would have done” (P6)

“What I have learned in the last year, 30 years into my nursing career is unbelievable”. (P11)

### 3.3. Theme 3. Barriers to the Development of the ANP Older Persons’ Role

Barriers may hinder the full utilisation of the ANP Older Persons’ role. The IDT identified three subthemes: Protection of the role, Governance and structure and Poor understanding of the role.

#### 3.3.1. Protection of the ANP Older Persons’ Role

Participants voiced concern about the undefined boundaries for the ANP Older Persons’ role. Acknowledgement of the ANP’s ability to provide high‐quality care in a challenging healthcare environment is admired by the participants; however, this may be detrimental to the progression of the ANP Older Persons’ role. Participants worry that the ANP may be lost within the health system if change does not occur;

I do feel, she feels a bit lost, you know, she’d maybe feel a bit lost and, you know, there’s not, probably not enough support out in the community for her. (P15)

At the minute they probably are a bit of a dumping ground for a lot of stuff. (P32)

*I just feel she’s been pulled in 10 different directions*… *I just think it’s too broad for one person*. (P11)

Participants highlighted the stressors and pressures within the role. The IDT has expressed worries regarding a *“lack of fitting in”* and a *“high demand may leave the ANP a bit stressed”* (P2). Participants fear that overtime, if support is not provided for the ANP Older Persons, the role will not develop:

I feel it’s quite a vulnerable position to be in. It’s a minefield at the moment. (P2)

I think that’s how you develop a role; you need to start with a very clear definition, it’s very stressful otherwise. (P7)

#### 3.3.2. Governance and Structure of the ANP Older Persons’ Role

Participants voiced concern over undefined boundaries and inconsistent governance, suggesting that ANPs are often left unsupported or misunderstood. Some ANPs were described as overwhelmed by competing responsibilities across settings. Participants expressed an emphasise on the importance of stakeholders from the conception of this role. P40 suggests the same:

From its conception the post was wrong because the stakeholders weren’t involved, and it never got set up then. (P40)

Participants voiced concerns regarding a lack of proper structure provided for the ANP Older Persons’ role or education about the role when first included in the IDT:

Well, I can say that when the role was brought in, it was very wishy‐washy… It kind of was set up in such a way that she could only kind of really fail. (P34)

For me, I just think that it’s not well enough known at the minute, you know, so I think they can get lost a little bit in the big scheme of things. (P17)

The ANP Older Persons have adapted well to adversities, but participants are demanding consistent, effective and vivid regulations that protect the ANP and allow for continuous advancements within the role:

In terms of governance, in terms of, you know, monitoring, making sure that the role stays as specific as it has been designed to be, that’s something that I think needs to be looked at. (P35)

I think it is important to have clear role descriptions and pathways and things like that, but to have flexibility within that to develop as services. (P4)

I think it needs a bit of clarity and maybe more education. It’s nearly like the structure is behind. (P36)

I think maybe a better definition of what she can do, because there’s only one of her, and there is the whole breadth and depth of things that she could do. (P9)

Participants also emphasised the importance of structured role introductions to the IDT, supported by leadership and ongoing education. More investment in the number of ANP Older Persons was deemed as a necessity within the IDTs’ clinical area:

I think you can afford to have an ANP who is focused on the ward and the one that’s focused on front facing, because there’s enough there for the front facing to be part of the team. (P9)

If there’s more ANPs in the community that didn’t have to get as far as an acute setting before they’re referred in that way, it would relieve the weightiness here. (P22)

It’s going to be difficult for ANPs, they should have more of them. (P34)

You can add on to the scope later, but you need to have a core kind of task that you would do, you know, whether it be clinical or education or research or whatever. (P9)

I think her role needs to be more, not more developed, but more out there, and maybe defined. I think they feel that too much time, too much of (ANP)’s role is being used in the acute services, rather than her role could be more beneficial in older persons. (P15)

Participants spoke of worries surrounding work boundaries with sharing tasks as the ANP Older Persons can overlap with many other clinical workers’ activities:

I think they need more autonomy because sometimes there’s that thing where, oh God, am I taking somebody else’s job, or are they taking my job, or am I stepping in somebody else’s toes? (P5)

#### 3.3.3. Poor Understanding of ANP Older Persons’ Role and Lack of Role Definition

Participant’s explanations of the ANP Older Persons’ role demonstrate that not all members may understand it;

I don’t think anything was explained no, and so I think I asked [ANP] myself… How do you even refer to or contact [ANP]…how do they keep a case? I don’t even know do they keep a caseload of patients? (P3)

I probably need to know more about the role and what exactly I can refer for instead of being like, I think that’s for you, I’m not sure if that’s for you. (P8)

We know that structure in terms of her line management and yeah, the framework over her work, that kind of framework could be a bit hazy. (P2)

Some participants admitted to never knowing the role had existed before the ANP Older Persons was part of their team (P22). A lack of organisation in the introduction of the ANP role to the IDT was evident from the participants. There was *“no opportunity for the ANPs to explain their role”* to the IDT (P36).

When I met her, I couldn’t tell you and did I know what her role entailed. (P11)

We don’t really know the background of how the posts came in. (P1)

Participants explained that due to a lack of role definition, the role was open to interpretation and had a flexible and inconsistent perception of the role with varying definitions. The IDT, however, agreed that the role is holistic, autonomous and provides episodic care;

It’s supposed to be more of a flexible role or obviously, it’s looking at a holistic view and patients and being able to provide episodic care. (P35)

It’s different here though, yeah, I think you’re getting that, like it’s much more defined here… She’s very much her own entity. (P32)

Many participants highlighted the fact that misunderstanding of the role may be due to its slow and new emergence within the IDT, and as the role becomes more established, there will likely be a greater understanding;

I just think that the role is pretty vast and new. I’m still learning about it. (P13)

It’s evolving naturally through working together rather than like a role description. (P4)

She would have just explained her role and explained that as it currently is, she’s finding her feet within this hospital too and what her role is. (P8)

**FIGURE 1:**
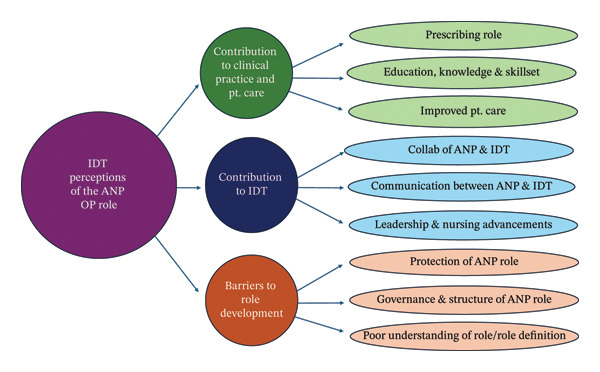
Themes and subthemes.

In summary, the emerging themes demonstrate that the ANP (Older Persons) plays a pivotal role in contributing significantly to clinical practice and patient care, as well as to collaboration and leadership within IDTs, while also encountering structural and governance‐related barriers to full role integration. The three overarching themes highlight both the breadth and complexity of the ANP (Older Persons) contribution to care delivery. Figure 1 presents a summary of the findings, outlining main themes and subthemes.

## 4. Discussion

This study explored the role of the ANP Older Persons from the perspective of the IDT. The findings demonstrate that the ANP Older Person makes substantial contributions to clinical practice and patient care, enhances team functioning and collaboration within IDTs and navigates a range of structural and governance‐related barriers that influence role development. These insights highlight both the complexity and the evolving nature of the ANP role, reflecting its growing integration into healthcare systems.

### 4.1. Theme 1: Contribution of the ANP Older Persons’ Role to Clinical Practice and Patient Care

The ANPs within this study had a significant role in clinical practice and patient outcomes. Positive aspects of this role included prescribing, education, knowledge and sharing of skillset, with subsequent improvements in patient care. According to IDT members, the presence of a specialised ANP has enhanced older persons’ care. This aligns with the study by Farrelly and Daly [19], who highlight that Older Persons’ experiences of frailty are multidimensional, encompassing physical, psychosocial and emotional domains, emphasising the need for holistic care approaches.

Although members of the IDT approach patient care differently due to specialised training and varied interventions, participants agreed that the role of the ANP Older Persons is advantageous for patient care. Patient care and patient flow management by ANPs was favoured in studies by Htay and Whitehead [20] and Sanson et al. [21], with similar findings emerging in this study. The positive aspects described by participants, including clinical leadership and person‐centred care, are consistent with the core domains of advanced nursing practice outlined in national standards [6], particularly clinical decision‐making and professional scholarship.

### 4.2. Theme 2: Contribution of the ANP Older Persons’ Role to the IDT

ANPs have adapted well to challenges within the IDT, as suggested in the literature by Cowley et al. [22], Kraus and DuBois [23] and Strachan et al. [24]. Although the role remains in its developmental stages, the benefits of the ANP Older Persons were well articulated by the members of the IDT. This reflects an ongoing process of professional role construction, whereby advanced nursing identity is negotiated relationally within clinical teams rather than solely defined through formal regulation.

Subtle differences in emphasis across professional groups were evident, with nursing participants more likely to highlight mentorship and professional development, while medical participants emphasised efficiency, workload redistribution and clinical decision‐making. This mirrors previous literature [22, 24, 25]. Participants referenced the ANP as reshaping expectations of the nursing role within interdisciplinary practice, providing task sharing and enhancement of care as a leader due to their unique strengths [26]. This may improve patient care and reduce workplace conflict [27].

Relationships between the ANP Older Persons and IDT members were described as strong and well established. Participants who had formed working relationships through shared care reported strong professional bonds, describing the ANP as open, approachable, knowledgeable and a reliable resource. Functional work relationships provide benefits for the clinical team [28] and facilitate high‐quality care [29]. The ANP Older Persons facilitate close professional relationships that support optimal role utilisation and care delivery.

### 4.3. Theme 3: Barriers to the Development of the ANP Older Persons’ Role

Despite these contributions, tensions emerged between role expectations and organisational realities, reflecting contradictions noted in the literature, including role ambiguity, overlapping responsibilities, and limited systemic support [19]. Persistent ambiguity surrounding the boundaries of advanced practice reflects a broader tension between professional autonomy and organisational control [8, 30].

IDT members valued the ANP role but identified challenges related to role clarity and definition. Participants highlighted the need for support and structure to enable continued role development, particularly as the ANP Older Persons’ role diversifies across clinical settings. Ongoing role evaluation and standardised induction were identified as essential [31]. Barriers included governance, structural requirements and limited stakeholder involvement [30, 32]. Participants suggested that these challenges may lessen as the role becomes more embedded within the IDT, with greater autonomy enabling increased investment and responsiveness to population needs [33, 34].

Whilst presented under three themes, the findings were closely interconnected. Leadership, advocacy and communication influenced clinical practice, patient care and collaboration across the IDT. The findings strongly correlate with competencies for advanced practice nursing [6], suggesting that despite boundary ambiguity, ANPs are operating within nationally defined domains and regulatory standards.

The integration of the ANP Older Persons’ role within the wider health system strongly influences care delivery and role effectiveness. Interprofessional understanding, role clarity and regular collaboration facilitated effective contribution, consistent with the findings by Porat‐Dahlerbruch et al. [8]. Conversely, inconsistent understanding or limited role definition hindered integration and role utilisation.

This study underscores the need for healthcare managers and policy stakeholders to implement structured governance frameworks and invest in leadership development for ANPs. Evidence‐informed induction, interdisciplinary education and performance feedback are essential [35]. These findings align with Sláintecare [36] and national policy on advanced practice development [5], emphasising the importance of investment in advanced nursing roles to meet the needs of an ageing population.

Throughout the study, the researcher recognised that their own background as a nurse may have shaped the interpretation of participant accounts, potentially emphasising clinical aspects of the ANP role over organisational or systemic challenges. Reflexivity was supported using Gibbs’ [37] framework, which guided reflection on personal biases, relationships with participants, methodological choices and the broader impact of the research; however, the researcher was aware of this in order to avoid bias. Gibbs’ six‐step cycle provided support during each phase of this study. These steps include description of the situation, feelings during the experience, evaluation of the situation, overall analysis, conclusion and an action plan. Gibbs’ framework harboured continuous objectivity from the researcher and ensured study data would not be tainted. Appendix IV presents the Gibbs reflective cycle (1988).

This study demonstrates that the ANP (Older Persons) plays a critical role across multiple domains, contributing significantly to clinical practice and patient care, enhancing collaboration and functioning within IDTs and navigating barriers to role development and integration. These findings underscore the complexity and evolving nature of the ANP role, highlighting its impact not only on patient outcomes but also on team dynamics and healthcare delivery. Importantly, this study is novel in exploring the ANP Older Persons’ role specifically from the perspective of the IDT, addressing a gap in the existing literature and providing insights unique to the older persons’ speciality. By capturing the perspectives of those who work closely with ANPs, this research offers a deeper understanding of role implementation, integration and potential strategies to support the continued development and sustainability of the ANP role in older persons’ care.

### 4.4. Study Limitations and Bias

Qualitative research may hinder the accuracy of data collected due to its emotive and descriptive nature [11]. Ensuring trustworthiness was a key consideration throughout the study [38]. Measures such as audio‐recording interviews, cross‐checking transcripts and systematically coding data were used to minimise recall bias and enhance credibility.

Selection bias may have occurred, as participants self‐selected to take part and were required to have experience working with an ANP Older Person. This may have influenced the predominance of positive perceptions reported. As data were generated in focus groups, the findings may have been influenced by social desirability and group dynamics, including professional hierarchies within IDTs. Formal member checking was not undertaken; however, credibility was supported through prolonged engagement with the data, reflexive analysis and transparent documentation of analytic decisions. Consistent with a reflexive thematic approach, intercoder reliability was not sought, as the meaning was coconstructed through researcher engagement with the data rather than consensus coding. Additionally, the researcher’s limited prior experience in this specific research context is acknowledged; while this may have reduced preconceptions, it may have posed challenges in fully anticipating contextual nuances. Finally, as this study was conducted within the Midlands Region of Ireland, the findings may not represent the experiences of the full ANP Older Persons’ network nationally, limiting broader transferability.

## 5. Conclusion

This study explores the role of the ANP Older Persons from the perspective of the IDT. Members of the IDT have highlighted the benefits brought to clinical care and patient outcomes by the ANP Older Persons, with all participants expressing the need for such a role in future healthcare. All participants stated that ANPs are a positive addition to nursing and healthcare and patient outcomes.

All participants highlighted that, overall, the ANP Older Persons’ role is developing rapidly and is making significant contribution to healthcare and services for older people. With the further development and establishment of the role, in the future the ANP Older Persons appear to be well placed to lead and deliver future healthcare.

Challenges and barriers raised in the study were predominantly about systems and structures in which advanced practice has been established. Undefined boundaries were perceived as a risk to the ANP Older Persons’ role. Lack of understanding and clarity of the role was linked to underutilisation of the skills of the ANP, thus optimising patient care. However, the findings are reassuring that ANPs are working within their professional boundaries.

### 5.1. Recommendations

• The study may benefit from replication nationally to obtain evidence as to how health professionals in other healthcare areas in Ireland perceive the role of the ANP Older Persons. Ongoing empirical data collection and policy change are needed to enable the full scope and strategic utilisation of ANP across healthcare systems and contexts. This warrants further research.
• This research was conducted over a year due to funding, so further research may be needed over a longer period of time.
• The ANP has had a positive effect on the care of the Older Person, colleague relationships and the development of professional upskilling. This role needs to be expanded and included in all acute and community sites including the integrated hubs, with a well‐structured format in place.
• Further investment in the number of ANP Older Persons available both within the Midlands and throughout Ireland will become a necessity to meet the demands of healthcare into the future. Continuous support and guidance provided by management will allow the role of the ANP Older Persons to become well established within the IDT. Consistent documentation used throughout the Midlands area for the ANP Older Persons may facilitate standardisation of work, allow for accurate review and renewal and assist clear communication amongst the IDT. Concerns regarding role definition and role boundaries remain a significant issue which will need to be addressed.
• Healthcare managers should collaborate with ANPs to codesign role frameworks and ensure sustained investment, supervision and leadership development opportunities.
• Ensuring sufficient investment in the role, fully expanded autonomy and scope of practice, standardisation in education and training and the encouragement of public awareness of the role, peer support and collaboration with healthcare members, will lead to the flourishment and proper establishment of the ANP Older Persons’ role.

## Author Contributions

A. Mullally: data curation, formal analysis, investigation, methodology, project administration, resources, software, visualisation, writing–original draft and writing–review and editing.

M. Doolan: conceptualisation, supervision, visualisation and writing–review and editing.

C. Brennan: resources, visualisation and writing–review and editing.

L. Burke: resources and visualisation.

A. Farrelly: conceptualisation, resources, validation, visualisation and writing–review and editing.

G. Flynn: resources, visualisation and writing–review and editing.

K. Heduvan: resources, validation, visualisation and writing–review and editing.

G. Keena: resources, project administration and visualisation.

O. Lynch: conceptualisation, project administration, resources, validation, visualisation and writing–review and editing.

J. Mannion: resources and visualisation.

F. Moore: resources and visualisation.

M. Nolan: conceptualisation, funding acquisition, resources and visualisation.

M. McDonnell‐Naughton: conceptualisation, formal analysis, funding acquisition, methodology, project administration, resources, supervision, validation, visualisation and writing–review and editing.

All authors were involved in submission of the research proposal seeking ethical approval. All authors reviewed the final manuscript.

## Funding

The study was funded by the Nursing and Midwifery Development Unit from the Health Services Executive for the President’s Doctoral Fund of the Technological University of the Shannon: Midlands and Midwest.

## Ethics Statement

This study was granted ethical approval by the Technological University of the Shannon: Midlands and Midwest, and ethical approval was also sought from the HSE and granted. This research was conducted in compliance with both their standards and regulations. Ethical approval number from the HSE is REECB0324AbM.

## Conflicts of Interest

This study was funded by the HSE, as mentioned previously in this application. The lead researcher for this study is paid for this position. This may be viewed as a conflict of interest, however the researcher and Scientific Advisory Group have approached this study fairly, so that this did not interfere with data or results of the study. This has not hindered the researchers’ objectivity on the topic of this study and therefore has not hindered the study outcome. The other authors declare no conflicts of interest.

## Figures and Tables

**Figure A1:**
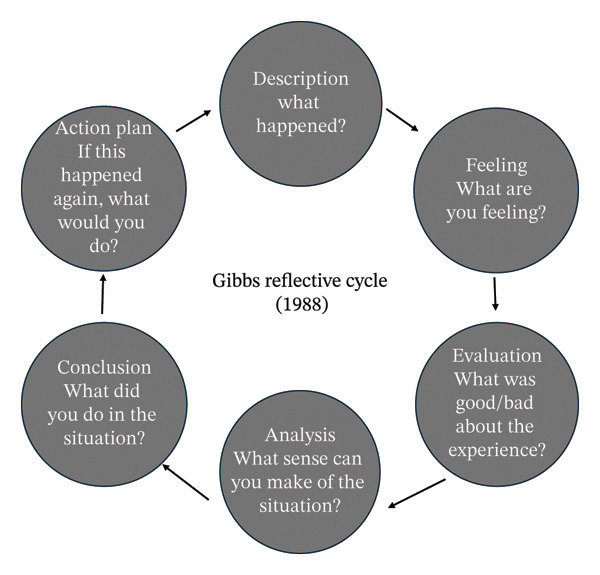
Visual representation of Gibbs′ reflective cycle [37].

## Data Availability

The data that support the findings of this study are available from the corresponding author upon reasonable request.

## Appendix I

**Table A1:** Six domains of advanced nursing practice [6].

Domain 1 Professional values and conduct of the registered advanced nurse practitioner	Domain 2 Clinical decision‐making	Domain 3 Knowledge and cognitive competences
Domain 4 Communication and interpersonal competences	Domain 5 Management and team competences	Domain 6 Leadership and professional scholarship competences

## 1 Appendix II

**Table A2:** Coding framework following the work of Braun and Clarke [12].

Initial codes	Subcodes	Final themes
Ability to prescribe	1.1 Prescribing role	*1. Contribution of the ANP Older Persons’ role to clinical practice and patient care*
Deprescribing		
Providing education to the IDT	1.2 Education, knowledge and skillset role	
Provide up‐to‐date training to the IDT		
“Fountain of knowledge”		
Older‐persons’ specific knowledge for the IDT		
Valuable skillset		
“Fills the gap” in care		
“Missing puzzle piece” in the IDT		
Effective communication		
Advocacy	1.3 Improved patient care	
Better quality of care		
Ageing population and demand		
New ideas for care		
Time wastage prevention		
Open communication and strong bonds	2.1 Collaboration between ANP older persons and IDT	*2. Contribution of the ANP Older Persons’ role to the IDT*
Well‐established work relationship prior to ANP OP role		
Effective communication skills		
Provides reassurance to IDT		
First port of call		
Works closely with majority of professionals		
ANP OP willing to share knowledge to IDT		
“Give and take” relationship		
Trust with ANP OP		
Reliability on ANP OP		
ANP OP as supportive model		
Co‐operative with IDT activities		
IDT felt no intimidation from ANP OP		
Approachable and accessible role		
IDT understand ANP’s unique position in team		
Strong relationship equates to strong understanding of role		
Role explained by ANP OP to IDT	2.2 Communication between IDT and ANP Older Persons	
IDT and ANP OP have open communication		
Seen as “middle ground” between nursing and medical framework		
Holistic and multifunctional role from ANP OP for the IDT		
Acknowledged progression of ANP OP in nursing field		
Expansion of nursing scope of practice	2.3 Leadership and advancements in nursing	
ANP OP seen as a leader		
Reignition of passion for nursing career		
Team reliance, respect and trust in ANP OP work for nursing team		
Role too broad	3.1 Protection of the ANP Older Persons’ role	*3. Barriers to the development of the ANP Older Persons’ role*
Lack of team investment in the ANP OP role		
Lack of ANP OP fitting in with IDT		
IDT protective of their profession		
Lack of structure within the ANP OP role		
Lack of consistency within ANP OP role description		
Lack of protection of ANP OP role		
ANP OP role blurring with other professions		
Lack of role boundaries		
Role pressures and stressors		
Role still establishing itself	3.2 Governance and structure of ANP Older Persons’ role	
Restriction to full autonomy and ability within the ANP OP role		
Lack of education about ANP OP role to IDT		
Improvement to policies, procedures, protocols and guidelines		
Lack of ANP OP positions		
Lack of role induction/explanation	3.3 Poor understanding of ANP OP role and lack of role definition	
Uncertain knowledge about ANP OP role		
Inaccurate understanding of ANP OP role		
Interchanging role definition		
Adaptive role definition		
Misinterpretation of ANP OP role		

## 2 Appendix III

**Table A3:** Semistructured topic guide for focus group.

Workload and colleague co‐operation	• Work relationship between ANP and IDT members • How often is contact with ANP made? • Overlap of work between ANP and IDT • ANP’s effect on colleague workload

Patient satisfaction and preference	• Patient re‐admission • Change to therapeutic relationship • Report of patient satisfaction from ANP care • IDT preference for ANP to provide patient care • Patient compliance with health requirements reported from IDT members • Communication between ANP and patients

Healthcare improvement	• Improved quality of care • How do ANPs effect healthcare? • Do IDT members think ANPs have improved care? • Do IDT members think the ANP role has benefited healthcare? • Are there disadvantages to the ANP role?

Career satisfaction	• Are ANPs enthusiastic within the workplace? • Have ANPs reported satisfaction with work? • Has the ANP changed nursing?

Colleague understanding of the role	• Are IDT members confident that they understand the ANP role? • Do IDT definitions of the role match actual role description? • Do IDT members feel that they misunderstand the role? • How was the role first introduced to IDT members? • ANP governance • ANPs or management explain the role to IDT?
